# Rickettsial Infections in Monkeys, Malaysia

**DOI:** 10.3201/eid2103.141457

**Published:** 2015-03

**Authors:** Sun Tee Tay, Fui Xian Koh, Kai Ling Kho, Frankie Thomas Sitam

**Affiliations:** University of Malaya, Kuala Lumpur, Malaysia (S.T. Tay, F.X. Koh, K.L. Kho);; Department of Wildlife and National Parks Peninsular Malaysia, Kuala Lumpur (F.T. Sitam)

**Keywords:** Rickettsia felis–like organism, Anaplasma bovis, Macaca fascicularis, bacteria, Malaysia, primates, monkeys, rickettsia

**To the Editor:** The cynomolgus monkey (*Macaca fascicularis*), also known as the long-tailed macaque or crab-eating monkey, is commonly found in the Southeast Asia region (*1*). The macaque has been associated with several bacterial infections, such as those caused by hemotropic *Mycoplasma* and *Bartonella quintana* (*2*). As a result of rapid deforestation and changes in land use patterns, cynomolgus monkeys live in close proximity to human-populated areas (*1*). Human–macaque conflict may increase the risk for zoonoses. 

Little is known about rickettsial and anaplasma infections in cynomolgus monkeys in Malaysia. Although *Rickettsia* spp. RF2125 and Rf31 have been identified from cat fleas in Malaysia (*3*), the presence of *Anaplasma bovis* in monkeys is not known.

*Rickettsia felis*, a member of the spotted fever group rickettsiae, is an emergent fleaborne human pathogen distributed worldwide (*4*). The obligate intracellular bacterium has been identified from cats, dogs, opossums, and the ectoparasites of various mammalian hosts. Several uncultured rickettsiae genetically closely related to the *R. felis*–type strain URRWXCal2 (referred to as *R. felis*–like organisms and including *Rickettsia* spp. RF2125, Rf31, *Candidatus* Rickettsia asemboensis, and others) have also been identified from various arthropods and fecal samples of primates (*5*). *A. bovis* is a gram-negative, pleomorphic, tickborne intracellular bacterium that infects a wide range of mammal species in many geographic regions (*6*).

To learn more about these infections in monkeys, we examined blood samples from 50 cynomolgus monkeys caught by the Department of Wildlife and National Parks at 12 residential areas in Peninsular Malaysia during a population management and wildlife disease surveillance program (January 2012–December 2013). Most monkeys (14 male, 36 female) were adults and were active and healthy. DNA was extracted from 200 μL of each blood sample by using a QIAamp DNA Mini Kit (QIAGEN, Hilden, Germany). We performed PCRs selective for the rickettsial citrate synthase gene (*gltA*) by using primers CS-78 and CS-323 and for the 135-kDa outer membrane protein B gene (*ompB*) by using primers 120-M59 and 120-807 (*7*). As positive controls, we used cloned PCR4-TOPO TA plasmids (Invitrogen, Carlsbad, CA, USA) with amplified *gltA* fragment from *R. honei* (strain TT118) and *ompB* fragment from a rickettsial endosymbiont (98% similarity to *R. raoultii*) of a tick sample. Amplification of anaplasma DNA was performed by using a group-specific primer pair (EHR 16SD/EHR 16SR) (*8*). As a positive control for the PCR, we used an *A. marginale–*infected cattle blood sample. The full-length sequences of the *Anaplasma* 16S rRNA gene were obtained by amplification with primers ATT062F and ATT062R (*9*). Sequence determination of the amplicons was performed by using forward and reverse primers of respective PCRs on an ABI PRISM 377 Genetic Analyzer (Applied Biosystems, Waltham, MA, USA). To search for homologous sequences in the GenBank database, we performed a BLAST (http://blast.ncbi.nlm.nih.gov/Blast.cgi) analysis and constructed a dendrogram based on 16S rDNA sequences of *A. bovis* (*10*).

The rickettsial *gltA* gene was detected from 12 (24%) blood samples of mostly male monkeys from 8 locations. BLAST analysis of 210 nucleotides (GenBank accession no. KP126803) amplified from all samples demonstrated 100% sequence similarity with *Rickettsia* sp. RF2125 (accession no. AF516333), *Candidatus* Rickettsia asemboensis (accession no. JN315968), and *Rickettsia* spp. clone 4G/JP102 and 11TP21 (accession nos. JN982949 and JN982950), which had been identified from cat fleas in Southeast Asia, Africa, and Costa Rica, respectively. The rickettsial sequence also showed 99.0% similarity (2-nt difference) with *R. felis–*type strain (accession no. CP000053). The rickettsial *ompB* gene was amplified from 4 samples, and BLAST analysis of the sequences (556–779 bp) revealed closest match to several *R. felis*–like organisms, including *Rickettsia* sp. RF2125 (100%, accession no. JX183538) and *Candidatus* Rickettsia asemboensis (99%, accession no. JN315972). BLAST analysis of the longest *ompB* sequence (accession no. KP126804) obtained in this study showed 93% similarity with that of the *R. felis–*type strain.

*Anaplasma* DNA was amplified from 5 (10%) monkeys at 2 locations by using group-specific primers. Analysis of the nearly full-length sequences of the *A. bovis* 16S rRNA gene (1,457 nt) revealed 3 sequence types (GenBank accession nos. KM114611–3) with 99.1%–99.2% homology to that of the *A. bovis* strain from cattle in South Africa (accession no. U03775). The phylogenetic tree (Figure) inferred by using various *Anaplasma* species confirms the clustering of the strains from monkeys with *A. bovis* from different animals (i.e., goats, cattle, deer, ticks, wild boars, dogs, raccoons, leopard cats, eastern rock sengis, and cottontail rabbits). Co-infection of *R. felis*–like organisms and *A. bovis* was detected in only 1 sample.

**Figure:**
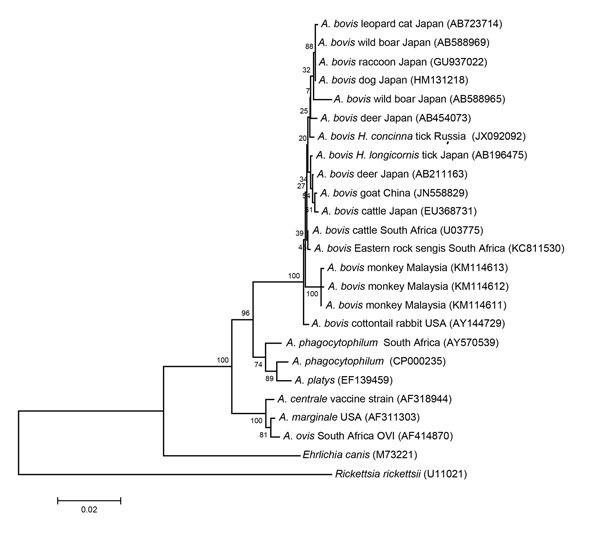
Phylogenetic relationships among various *Anaplasma* species, based on partial sequences of the 16S rRNA gene (1,263 bp). The dendrogram was constructed by using the neighbor-joining method in MEGA6 software (*10*) with the maximum composite likelihood substitution model and bootstrapping with 1,000 replicates. *Rickettsia rickettsii* (U11021) was used as an outgroup. Numbers in brackets are GenBank accession numbers. Representative Malaysian *A. bovis* sequences were deposited into the GenBank database under accession nos. KM114611–3. Scale bar indicates nucleotide substitutions per site.

Infections caused by *R. felis*–like organisms and *A. bovis* in the cynomolgus monkeys were subclinical (i.e., monkeys showed no evident signs of infection at the time of blood sampling). The diverse range of the organisms’ ectoparasite and animal hosts raises concern about their potential risk to human and animal health. Further study on the interactions between the microbes, vectors, and reservoir hosts is needed to assess their effects on public health.
